# Predicting Structural Properties of Pure Silica Zeolites Using Deep Neural Network Potentials

**DOI:** 10.1021/acs.jpcc.2c08429

**Published:** 2023-01-13

**Authors:** Tyler G. Sours, Ambarish R. Kulkarni

**Affiliations:** Department of Chemical Engineering, University of California, Davis, Davis, California95616, United States

## Abstract

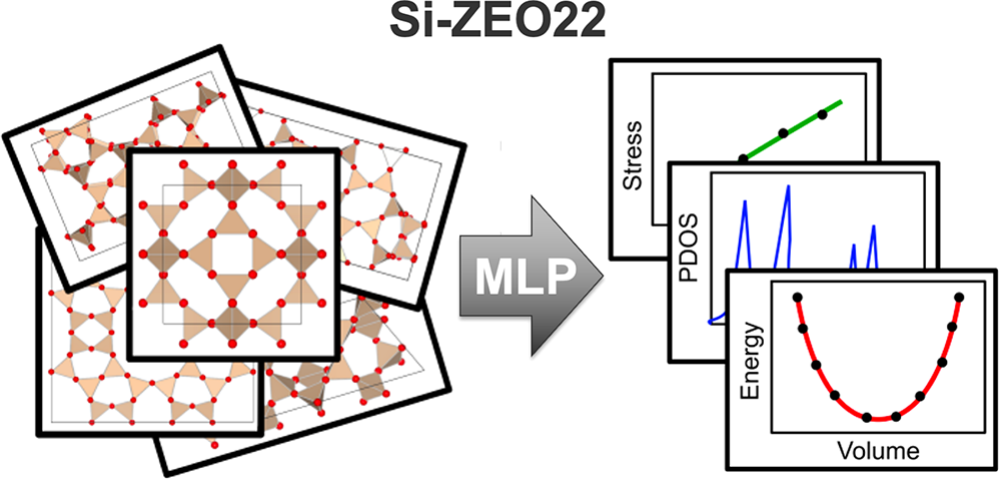

Machine learning potentials (MLPs) capable of accurately describing complex *ab initio* potential energy surfaces (PESs) have revolutionized the field of multiscale atomistic modeling. In this work, using an extensive density functional theory (DFT) data set (denoted as Si-ZEO22) consisting of 219 unique zeolite topologies (350,000 unique DFT calculations) found in the International Zeolite Association (IZA) database, we have trained a DeePMD-kit MLP to model the dynamics of silica frameworks. The performance of our model is evaluated by calculating various properties that probe the accuracy of the energy and force predictions. This MLP demonstrates impressive agreement with DFT for predicting zeolite structural properties, energy–volume trends, and phonon density of states. Furthermore, our model achieves reasonable predictions for stress–strain relationships without including DFT stress data during training. These results highlight the ability of MLPs to capture the flexibility of zeolite frameworks and motivate further MLP development for nanoporous materials with near-*ab initio* accuracy.

## Introduction

Accurate and efficient calculation of the energies and forces of atomistic systems remains one of the leading challenges in computational chemistry. While *ab initio* approaches rooted in quantum mechanics, e.g., density functional theory (DFT), often yield reliable results, large scale simulation of system dynamics with DFT remains impractical. For instance, predicting self-diffusivity coefficients, phase transitions, and phonon spectra using molecular dynamics (MD) often requires millions of force and energy evaluations. Traditionally, generic or DFT-parametrized force fields (FFs) are used for such computationally demanding simulations. While the simplicity of the FF methods enables longer simulation time scales for larger systems, these approaches are often less accurate than *ab initio* simulations. Even for FFs derived from DFT calculations, the rigid analytical form of bonded (e.g., harmonic, Morse, etc.) and nonbonded (e.g., 12–6 Lennard-Jones, Buckingham, etc.) potentials often results in systematic deviations.^1^

In contrast to the simple analytical form of classical FFs, machine learning potentials (MLPs) have emerged as a flexible alternative to describe complex potential energy surfaces. Specifically, by training the model on a suitable set of first-principles data that spans the relevant configuration space of a system, an MLP is able to evaluate the potential energy surfaces (PESs) at accuracy close to the *ab initio* method at significantly lower computational cost. Several different MLP forms have been proposed, which are broadly classified as either kernel methods or neural network methods. Kernel methods, such as GAP^2^ and sGDML,^3^ employ kernel functions (e.g., SOAP^4^) to assess the similarity of atomic configurations and interpolate the energy from known data points. Neural network methods calculate single atomic energy contributions by using a set of symmetry invariant descriptors that capture the local environment of each atom as inputs to various neural network architectures. Popular neural network potentials include ANI^5^ and DeePMD^6,7^ and newer message-passing networks like PhysNet,^8^ SchNet,^9^ and SpookyNet.^10^ New MLPs continue to appear in the literature, and several reviews exist describing and comparing the current state of the art models.^11−15^

Open-source releases of MLP software have enabled researchers to develop their own force fields for various systems including small molecules, nanoparticles, and metal surfaces. However, to the best of our knowledge, similar approaches have not been used for zeolites. Siliceous zeolites are polymorphs composed of the SiO_2_ formula unit with significant industrial use.^16,17^ Given the chemical simplicity and the existence of over 200 unique topologies and hundreds of thousands of theoretical structures,^18^ zeolites are ideally suited for demonstrating the capabilities of MLPs.

Many industrially relevant applications of zeolites involve small molecules diffusing through the porous framework over relatively long time scales. As including framework flexibility is necessary to accurately model diffusion and adsorption phenomena in zeolites,^19,20^ it is important to develop MLPs that accurately model dynamics of the framework. Thus, the central goals of this work are to develop a DFT data set that rigorously samples the atomic configuration space of pure silica zeolites and train and validate an MLP using the Deep Potential (DP) method implemented in DeePMD-kit.^7^

The DP method represents the system energy as the sum of single atomic energies that are determined from descriptors that capture the localized interactions between each atom and its neighbors within a specified cutoff distance. For a given atom, the relative coordinates of the local environment (i.e., the neighboring atoms) are passed through an encoding network to obtain symmetry invariant descriptors. These descriptors are then mapped to single atomic energies via an additional fitting neural network. This approach has shown promising results for describing the dynamics of both small molecules^21−25^ and periodic bulk materials.^26−31^ Additionally, the DeePMD-kit provides seamless integration with the Atomic Simulation Environment (ASE),^32^ the Large-scale Atomic/Molecular Massively Parallel Simulator (LAMMPS),^33^ and several other popular molecular simulation platforms.

In this work, a large DFT data set is generated using 219 of the 248 siliceous zeolite topologies found in the International Zeolite Association (IZA) database; all topologies with fully connected frameworks and less than 400 atoms were included. A single DP model, trained on 187 of these topologies to obtain a generalized silica MLP, is shown to accurately predict energies and forces of DFT configurations not included in the training set. This analysis is extended by using our DP model to calculate properties not explicitly included in the training, and the results are compared with the DFT predictions. Our results show excellent agreement between DP and DFT for structural properties, equations of state (EOS), and phonon density of states (PDOS). We also demonstrate the ability of DP to model stress–strain behavior and give reasonable predictions of mechanical properties even when *ab initio* stress data are not used during training. While other ML models have been developed to predict some of these properties purely from zeolite geometric descriptors,^34−36^ we test how well a DFT-trained MLP can directly calculate these properties by evaluating the PES. Our results are also compared with those from the BKS force field^37^ (used as a prototypical example of a classical force field), and we find that the DP model provides significantly more accurate results. We end our analysis by calculating the above properties for an additional set of 32 topologies (not included in the training) to demonstrate the transferability of the model. Taken together, by highlighting the efficacy of the DeePMD-kit formulation for silica zeolites, this study lays a foundation for future exploration of more complex materials such as those containing extra-framework cations and adsorbates.

## Computational Methods

### Training Set Generation

**Figure 1:**
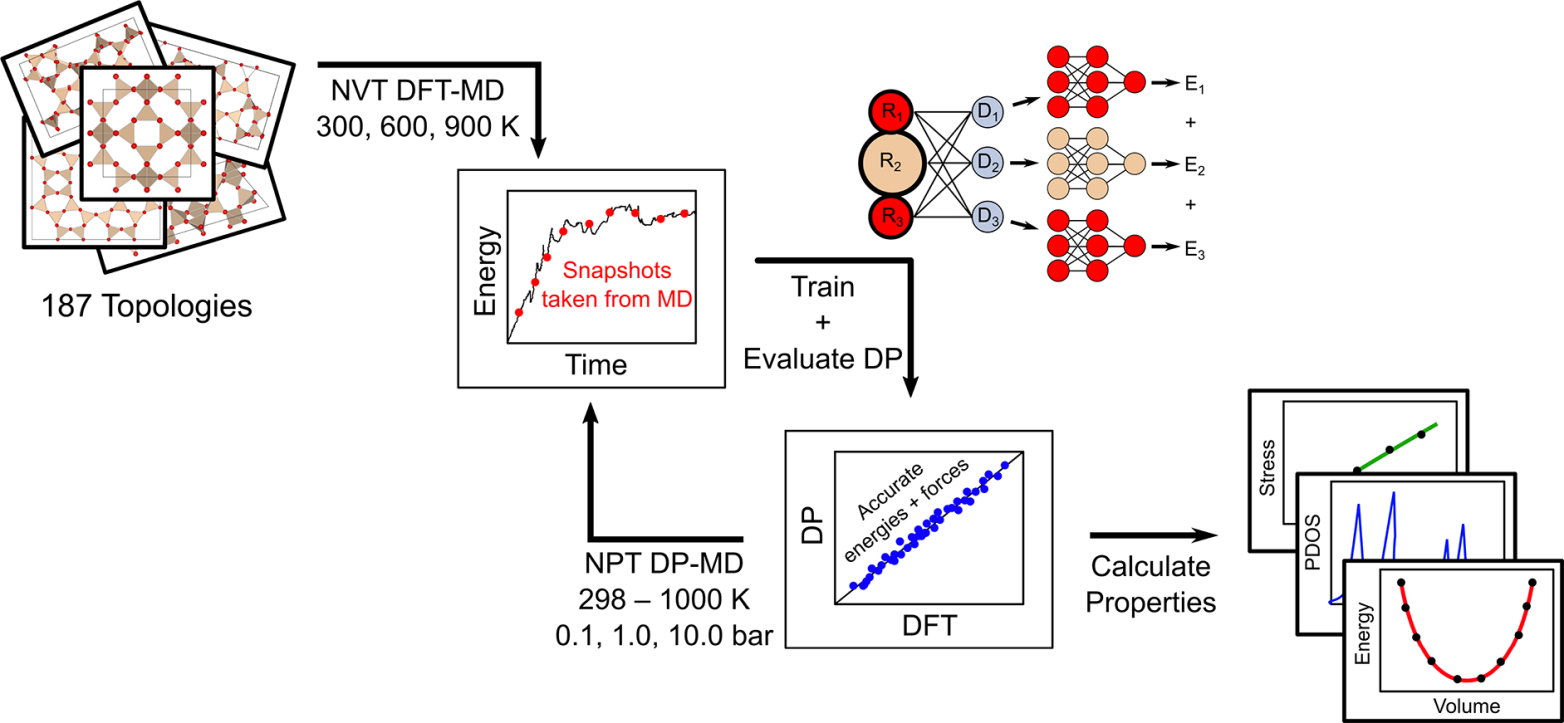
Schematic overview of the procedure used to train the DP model. The initial model was trained on configurations from 3 ps NVT DFT-MD runs at 300, 600, and 900 K. The initial DP was then used to generate 100 ps NPT DP-MD trajectories at pressures of 0.1, 1.0, and 10.0 bar with the temperature linearly ramped from 298 to 1000 K. Snapshots from every 1000 time steps were selected to obtain new uncorrelated configurations for training the final DP model that is used to predict various structural properties of silica zeolites.

DFT NVT-MD was used to generate the initial training set for the DP model. The Vienna *ab initio* simulation package (VASP) was used with the PBE^38^ functional for DFT calculations. Dispersion corrections were considered with the DFT-D3 method with Becke–Johnson damping (D3BJ).^39−41^ Only the Γ-point was used for k-space sampling. A plane-wave cutoff of 400 eV was used, and electronic energies were converged to 10^–5^ eV. Configurations were obtained from MD trajectories (≥1.5 ps simulation time, 0.5 fs time step) at three different temperatures: 300, 600, and 900 K. Snapshots from these trajectories were taken every 10 time steps and were used to train an initial DP model. This model was then used with LAMMPS to generate 100 ps NPT-MD trajectories at 0.1, 1.0, and 10.0 bar. After equilibrating the system (298 K, 10 ps), the temperature was ramped from 298 to 1000 K over the course of the simulation. This approach provides a diverse set of configurations at various temperatures and pressures. Snapshots of each system at every 1000 time steps were extracted for a total of 600 configurations (200 × 3 pressures). The energies and forces of the new configurations were evaluated with DFT, and the model was retrained including these results. This procedure is illustrated in Figure 1. Due to computational cost, fewer configurations were collected for large unit cell topologies; a full list of the topologies and corresponding data set sizes are included in the Supporting Information. While not used in this work, we note that the DPGEN^42,43^ training protocol can be used to select snapshots for training.

### Model Training

The configurations collected from each run were first shuffled and then split into 80% training data, 10% validation data used by the DeePMD-kit during the training process, and an additional 10% testing data. The cutoff radius is 6.0 Å with smoothing beginning at 5.5 Å. The embedding net was set to 3 layers with 16, 32, and 64 neurons, respectively. The fitting net was also set to 3 layers with 64, 64, and 64 neurons. The model was trained for 2 × 10^7^ steps with the learning rate starting at 5 × 10^–4^ and exponentially decaying to 5 × 10^–8^. The prefactors for the energy and force contributions to the loss function were set to *p*_*e*_^*start*^ = 0.02, *p*_*e*_^*limit*^ = 1, *p*_*f*_^*start*^ = 1000, and *p*_*f*_^*limit*^ = 1. Validation set learning curves for several model architectures are shown in Figures S1–S5. The hyperparameters selected were found to provide a reasonable balance between accuracy and training/evaluation time (Figure S6). The complete input file of all parameters used for training the final model is included in the Supporting Information.

## Results and Discussion

### Model Performance

**Figure 2:**
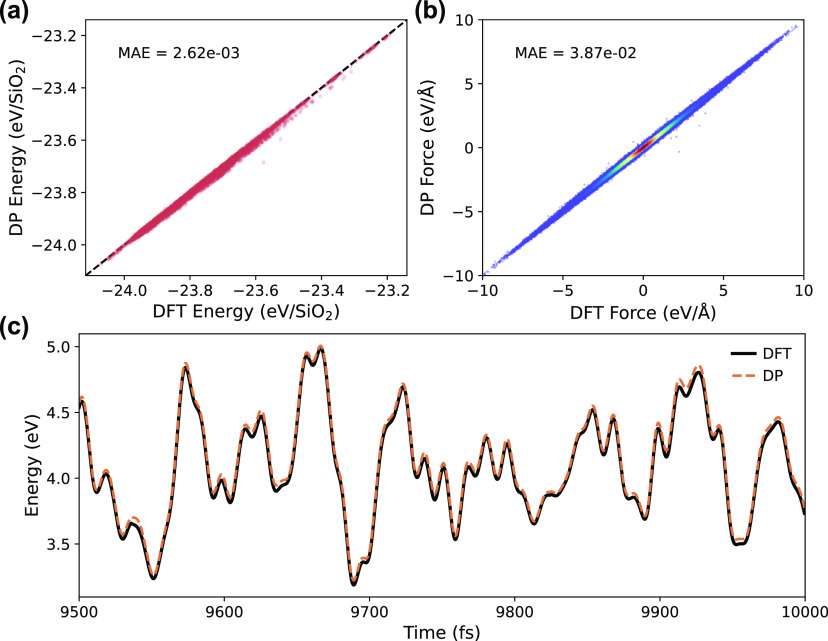
Parity plots comparing DP-predicted (a) energies and (b) forces with corresponding DFT values for the test data set not seen during training. (c) Energy relative to the relaxed structure from the final 500 fs of a 10,000 fs DFT-MD (solid black line) run with DP predictions overlaid (dashed orange line) for CHA topology at 298 K.

The accuracy of the energy and force predictions for the trained DP model were evaluated using testing data that was unseen during model training (10% of the original data set for each topology was set aside for post-training testing). The parity plots comparing DP predictions to DFT values for the energies (per SiO_2_ unit) and forces are shown in Figure 2a,b, respectively. DP was found to be able to predict DFT values with excellent accuracy, as seen by the MAE of 2.6 × 10^–3^ eV/SiO_2_ for energies and 3.9 × 10^–2^ eV/Å for forces. Note that the data shown in Figure 2a,b corresponds to the combined test sets of all 187 training topologies considered. Predictions for some topologies were found to be more or less accurate than others, and the complete list of MAE values for all individual topology test sets is included in the Supporting Information.

To further demonstrate our DP’s ability to predict energies and forces on configurations outside of the training set and to probe for any potential sampling biases arising from only including short DFT-MD trajectories in the initial training set, an additional 20,000 step DFT-MD run at 298 K was completed for CHA topology. The energies and forces of all configurations of the trajectory were evaluated with DP, and the MAE for the entire trajectory was found to be 0.95 × 10^–4^ eV/SiO_2_ for the energy and 2.0 × 10^–2^ eV/Å for the force predictions. Figure 2c shows the DFT energies (black line) of the final 500 fs snippet from the trajectory with DP evaluations overlaid (dashed orange line).

### Structural Properties

**Figure 3:**
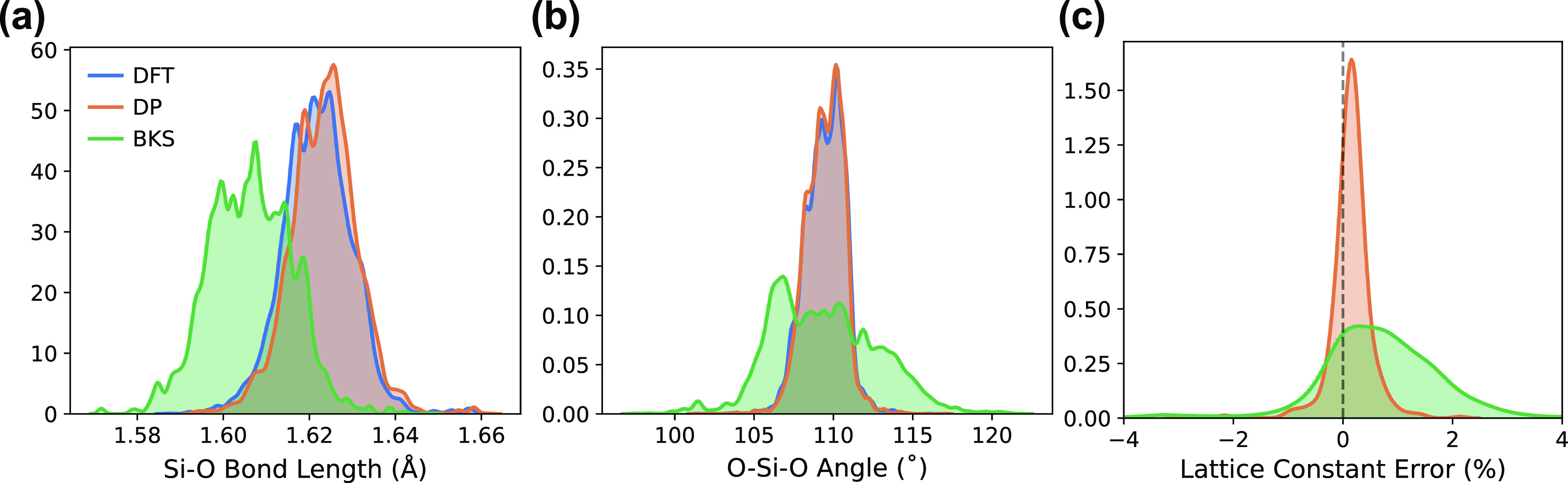
Normalized distributions of (a) Si–O bond lengths and (b) O–Si–O angles for relaxed geometries of the 187 topologies included in the training set for DFT, DP, and BKS. (c) Normalized distribution of percent errors relative to DFT of optimized lattice constants for DP and BKS. Vertical dashed black line denotes zero error (perfect agreement with DFT lattice constant).

The structures of all 187 topologies were relaxed using DP and compared to DFT optimizations. The normalized distribution of all Si–O bond lengths and O–Si–O angles for all relaxed structures is shown in Figure 3a,b, respectively. The distributions for both angles and bond lengths with DP match almost perfectly with the DFT distributions, highlighting the remarkable ability of DP to replicate relaxed *ab initio* geometries.

The percent error distributions in calculated lattice constants relative to DFT values for DP (orange) and BKS (green) are shown in Figure 3c, where positive and negative errors correspond to overestimation and underestimation of lattice constants, respectively. The narrow distribution centered at 0% error for DP implies excellent agreement with DFT. BKS shows a wider distribution centered at positive error, indicating a slight tendency to overestimate the lattice constants compared to DFT. These results show that a DP trained on higher energy MD snapshots can still produce very similar global minima to the DFT PES.

We acknowledge that recently reported classical zeolite force fields^44,45^ may show better performance than the BKS model used in Figure 3. As the central goal of this study is to develop a MLP model that shows similar accuracy to the DFT data, the comparison with other classical force fields (beyond the BKS model) is beyond the scope of this work. Interested readers are referred to the seminal work of Sastre for more in-depth comparison across different zeolite force fields.^20,46,47^

### Equation of State

Energy versus volume curves at 0 K were generated with DFT, DP, and BKS to assess how well DP can predict energies of systematically varied cell volumes. The resulting data were fit to the third-order Birch–Murnaghan EOS,
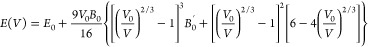
where *E*_0_ and *V*_0_ are the energy and volume of the relaxed structure, respectively, and *B*_0_ and *B*_0_^′^ are the bulk modulus (a property that describes the resistance to uniform compression/expansion) and its derivative. Thus, the bulk modulus can be determined from fitting energy–volume data to an EOS and serves as an additional metric for evaluating the performance of DP.

**Figure 4:**
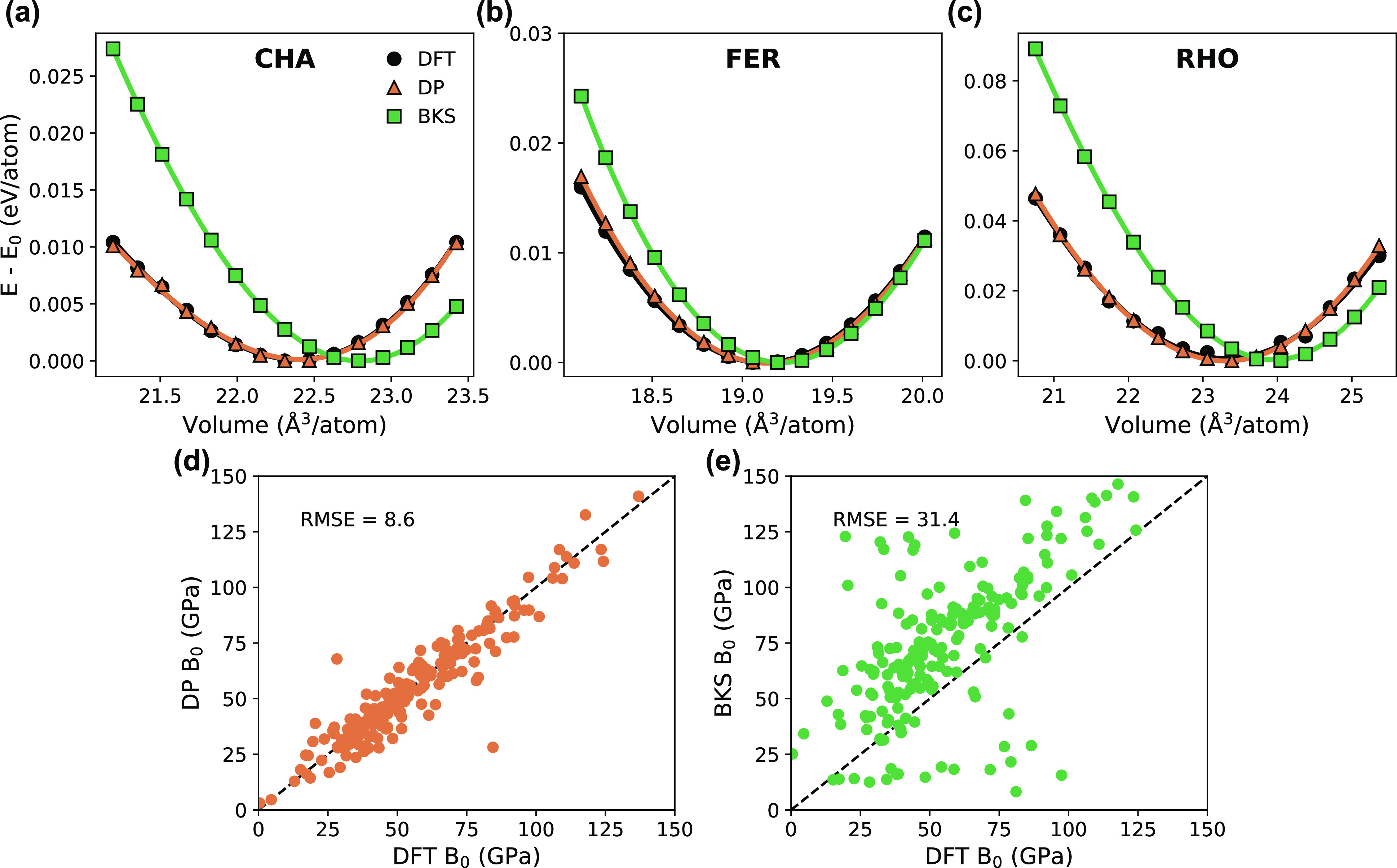
Energy–volume curves with third-order Birch–Murnaghan EOS fit for (a) CHA, (b) FER, and (c) RHO topologies for 15 volumes across ±5% volumetric strain. Parity plots comparing DFT with (d) DP and (e) BKS for bulk moduli calculated from EOS fits for all 187 topologies using 5 volumes across ±2% volumetric strain.

Taking the topologies of CHA, FER, and RHO as examples, the energy–volume curves and EOS fits are shown in Figure 4 for 15 volumes across ±5% volumetric strain. The DP data aligns very well with DFT, while BKS noticeably deviates. The similar curvature of the EOS fits for DP and DFT suggests DP can accurately calculate bulk moduli. Additionally, the similar location of *V*_0_ (the volume corresponding to the minimum energy) is further evidence that DP can accurately predict lattice constants. The higher curvature of the BKS energy–volume data implies that BKS overestimates the bulk moduli, and similarly, the values of *V*_0_ imply BKS overestimates the lattice constants for these topologies.

We extended this analysis to all other topologies included in the data set, and the resulting parity plots comparing predicted bulk moduli values with DFT values for DP and BKS are shown in Figure 4d,e, respectively. For computational efficiency, only 5 volumes were used with ±2% volumetric strain. The RMSE of bulk moduli calculated with DP was found to be 8.6 GPa, while BKS values had an RMSE of 31.4 GPa. Again, we see that BKS has a tendency to overestimate the bulk moduli in comparison to DFT.

### Mechanical Properties

**Figure 5:**
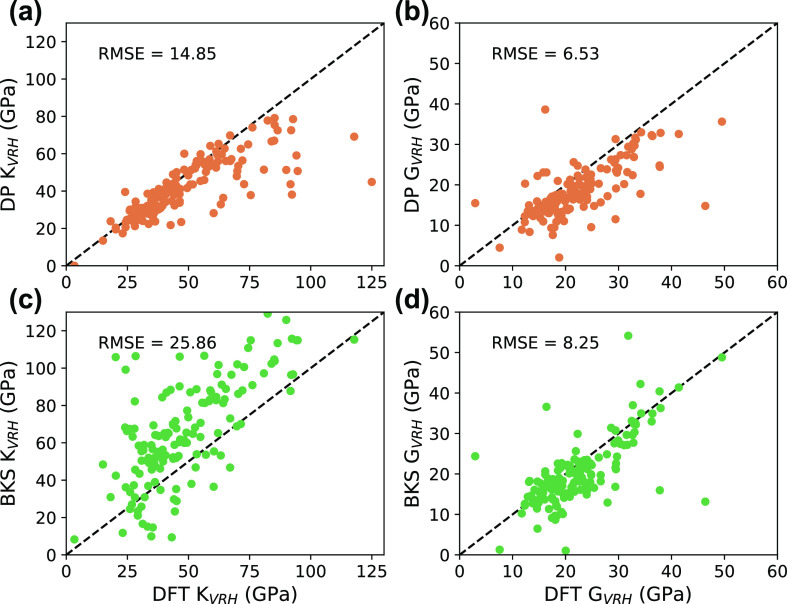
Parity plots comparing DFT VRH averages with DP-calculated (a) bulk moduli and (b) shear moduli and BKS-calculated (c) bulk moduli and (d) shear moduli.

Second-order elastic constants were calculated with Elastool^48^ using the optimized high-efficiency strain-matrix set (OHESS) using 5 strains (±2% amplitude) for each deformation.^49^ The elastic constants were used to compute Voigt-Reuss-Hill (VRH) averages of the bulk (*K*_*VRH*_) and shear (*G*_*VRH*_) moduli for 172 topologies. Figure 5 shows the agreement of DP and BKS with DFT for *K*_*VRH*_ and *G*_*VRH*_. DP is able to predict *K*_*VRH*_ quite accurately for topologies with values less than around 60 GPa; however, there is a noticeable drop in accuracy for stiffer materials with high *K*_*VRH*_ values, with DP consistently underestimating bulk moduli relative to DFT. This suggests that our DP model struggles to reproduce the expected stress–strain behavior for stiff topologies with high stress tensor values. Additionally, while DP tends to underestimate *G*_*VRH*_, the overall predictions are comparable to the BKS predictions.

Accurate calculation of elastic constants using stress–strain relations requires accurate stresses, so the DFT calculated bulk and shear moduli were calculated using a 700 eV plane-wave cutoff to ensure convergence of the stress tensor components. We note that it is possible to train a DP model including virial stress error in the loss function, and doing so would likely improve the accuracy of the mechanical property calculations. However, the DFT training set configurations were calculated using a plane-wave energy cutoff of 400 eV, and higher cutoffs are needed to converge the stress tensor components. Therefore, it would not be appropriate to use the stress values for training. Notwithstanding these limitations, it is quite impressive that DP can produce reasonable predictions of mechanical properties that were calculated with a 700 eV cutoff even though the training data consists entirely of configurations calculated at 400 eV. We note that it is necessary to include stress data in the training and ensure the appropriate basis set is used to give reliable stress tensors to train on, as shown by the accurate calculations obtained in other work.^30^ We also note that better agreement may be obtained by using methods that calculate elastic constants from energy–strain relationships as opposed to stress–strain. However, a detailed investigation into mechanical properties is beyond the scope of this work, and we elected to use stress–strain approaches to examine the accuracy of DP-calculated stresses when not included in training.

### Phonon Density of States

**Figure 6:**
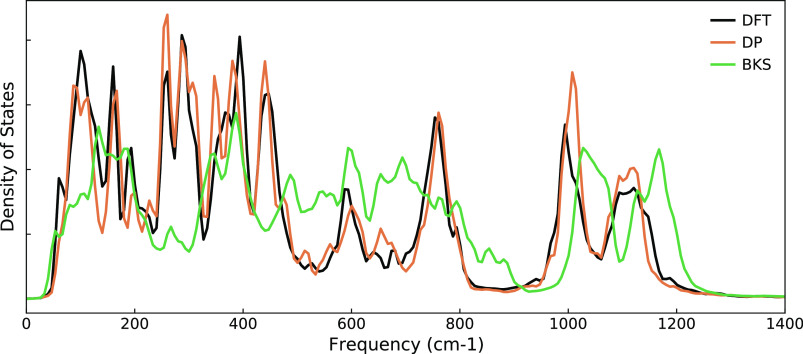
PDOS of CHA at 900 K calculated from the velocity autocorrelation function from an NVT-MD trajectory for DFT, DP, and BKS.

The PDOS of CHA (chosen due to lower DFT computational cost) was calculated at 900 K to assess DP’s ability to calculate vibrational modes. Atomic velocities from MD trajectories were used to calculate PDOS from the Fast Fourier transform of the velocity autocorrelation function. An MD trajectory of 10 ps was used for the DFT PDOS (black line in Figure 6), and 50 ps was used for DP and BKS (orange and green lines, respectively) PDOS calculations. As the PDOS is calculated from the changes in atomic positions, which are determined by the atomic force calculations at each MD step, it provides a good metric to probe the accuracy of the DP forces. We see good agreement in the frequencies of the vibrational modes between DFT and DP, while the intensity of the peaks is generally consistent but with some disagreement at a few frequencies. BKS shows a tendency to overpredict vibrational mode frequencies with broader and less intense peaks. These data demonstrate the suitability of the DP model for predicting phonon modes of silica zeolites at close to DFT accuracy.

### Model Transferability

**Figure 7:**
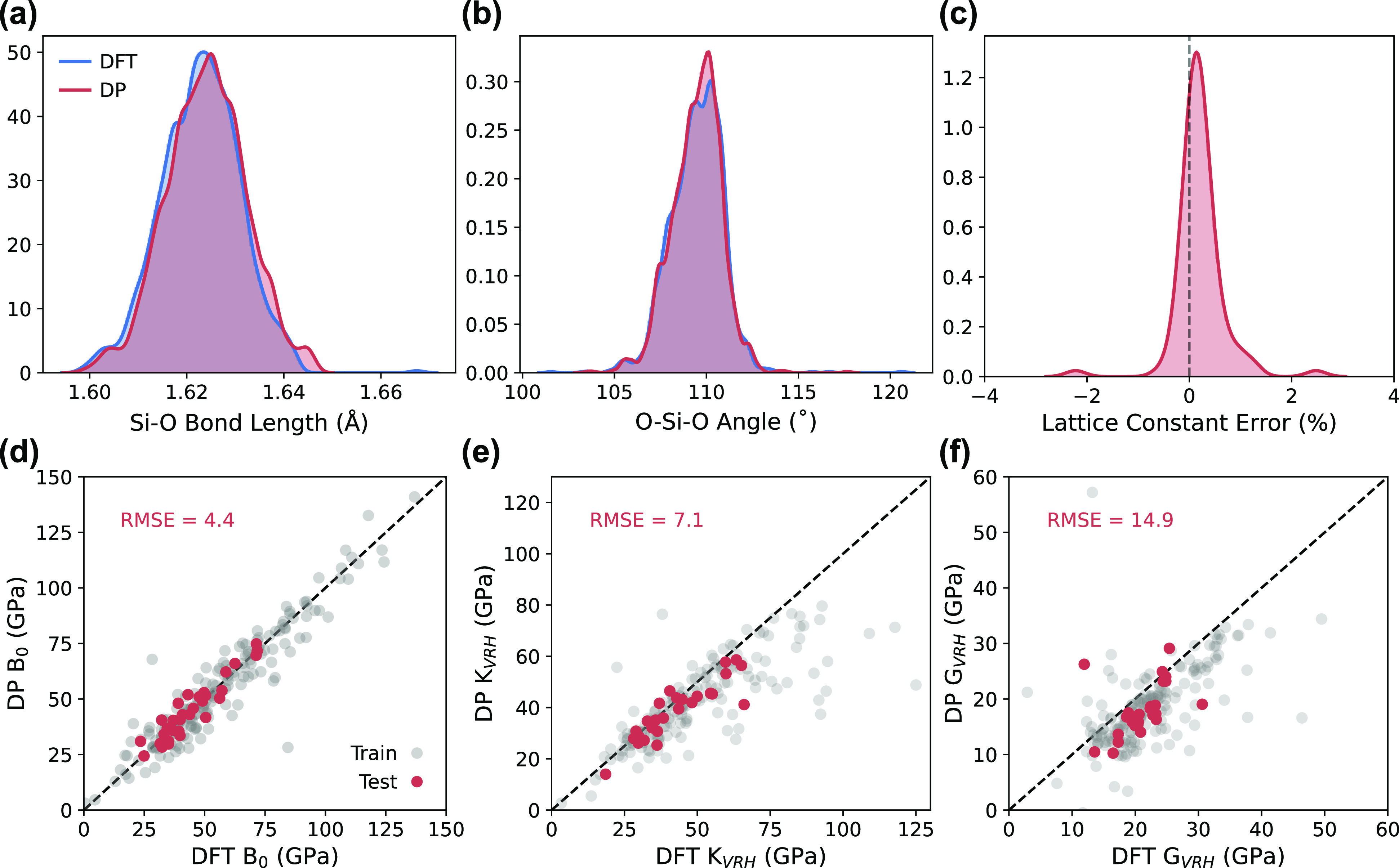
Comparison of normalized distributions of (a) Si–O bond lengths and (b) O–Si–O angles for optimized DFT and DP geometries of 32 topologies foreign to the trained model. (c) Normalized distribution of percent errors relative to DFT of optimized lattice constants for DP. (d) Bulk moduli calculated from EOS fits, and (e) bulk moduli and (f) shear moduli calculated from stress–strain relationships for the 32 testing topologies (red) compared to the 187 topologies included in training (gray).

The previous results assessed DP’s ability to predict properties of the 187 topologies included in the model’s training. We now examine a testing set of 32 topologies from our data set (not used during training) to see how DP performs for topologies completely unseen by our model. The optimized geometries of these 32 zeolites were obtained using both DFT and our DP model to assess DP’s ability to predict PES minima for new topologies. As seen in Figure 7a–c, the DP model continues to show impressive agreement with DFT for optimized geometries of new topologies. Both the optimized Si–O bond length and O–Si–O angle distributions align almost perfectly with DFT, and the calculated lattice constants agree with DFT typically within 1% error for the majority of topologies considered.

The calculations of bulk moduli (*B*_0_) from fitting energy–volume data and bulk and shear moduli (*K*_*VRH*_ and *G*_*VRH*_, respectively) from elastic constants using stress–strain data were repeated for the test set of topologies. As shown by the excellent agreement between DP and DFT calculated EOS bulk moduli in Figure 7d, we find our DP is transferable and capable of mapping out PES of unseen topologies by learning the PES of many similar structures. High transferability suggests that MLPs may be ideally suited for applications involving high-throughput screening of large zeolite databases by calculating a property of interest at near-DFT accuracy. The DP model also yields reasonable *K*_*VRH*_ (Figure 7e) and *G*_*VRH*_ (Figure 7f) predictions for the testing topologies with an accuracy on-par with that obtained for the training topologies. We reiterate that DFT stresses were not included in training, so it should be expected that *K*_*VRH*_ and *G*_*VRH*_ (calculated using stresses) are less accurate than *B*_0_ (calculated using energies) for both the testing and training topologies.

### Computational Cost

**Figure 8:**
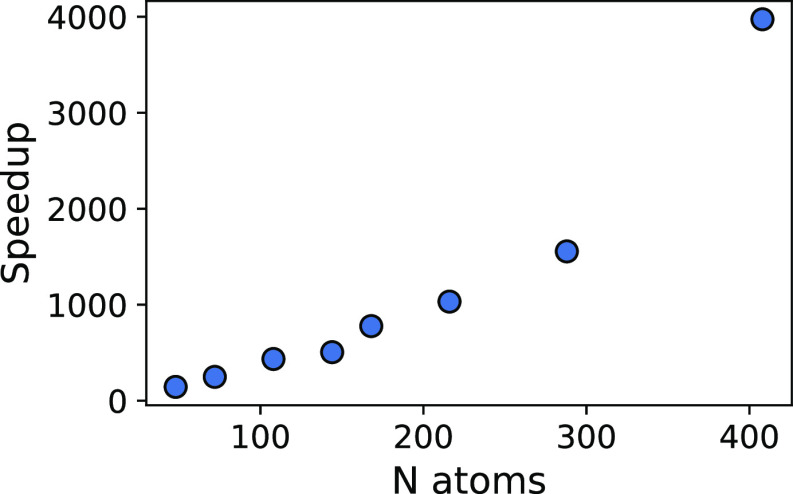
Computational speedup and scaling for DP compared to DFT for various silica topologies. The eight topologies chosen correspond to ACO, GME, CHA, MOR, SAO, STI, MFI, and IWS in order of increasing number of atoms.

We end our discussion with a brief analysis of the computational efficiency of DFT and DP. The average time per MD step was found for DP and DFT for eight randomly selected topologies of varying system size. The DFT and DP calculations were both performed using 32 cores (2.3 GHz Intel Xeon Processor E5-2698 v3) for a direct comparison of performance. Although using a fixed number of cores neglects potential scaling differences between DP and DFT with increasing CPU cores, an exhaustive cost analysis across different parallelization schemes is beyond the scope of this work. Figure 8 shows the speedup (ratio of DFT and DP time per MD step) for increasing system size. For our pure silica zeolite systems, we found DP to be >1000 times faster than the corresponding DFT calculation, with more favorable scaling of DP with an increasing number of atoms leading to improved speedup for larger systems. Coupled with the accuracy of the results discussed previously, we conclude that the DP approach significantly improves the accuracy–cost trade off for these materials. Note that the above results were obtained with the CPU version of DeePMD-kit; using GPUs could lead to better performance and improved parallelization.

## Conclusion

In this work, a diverse DFT data set was generated consisting of 219 pure silica zeolite topologies for training MLPs. DeePMD-kit was used to train a single DP on 187 of the 219 topologies (32 were set aside as a test set for model transferability) that accurately reproduces the *ab initio* PES of silica. We assessed the ability of the DP to calculate properties that were not explicitly trained for through energy and force evaluations. We have shown excellent agreement with DFT structural properties, as seen by nearly identical tetrahedral SiO_4_ geometry and lattice constants in structures relaxed by DFT and DP. The accuracy of the energies and forces was also highlighted by good agreement with DP and DFT for energy–volume curves (EOS) and finite temperature PDOS calculated from MD velocities. Mechanical properties from elastic constants calculated from stress–strain relationships were found to show reasonable agreement, with large improvement likely to be gained from including DFT stresses during training. We also tested how our model performs at calculating these same properties for the 32 testing topologies not included during training, and we found comparable accuracy to the training set topologies, suggesting a generalized DP applicable for any pure silica zeolite structure. Our results indicate an MLP trained on *ab initio* data can successfully model zeolite framework dynamics. We are currently extending the DP approach to model the diffusion of small molecules and metal nanoclusters in zeolites and metal–organic frameworks (MOFs). Our findings provide a promising avenue to develop DP-based MLPs for zeolites and are broadly relevant to the nanoporous modeling community. Additionally, we anticipate that the silica zeolite data set developed in this work (denoted as Si-ZEO22) will motivate the development of other MLPs for this important class of industrially relevant materials.

## Data Availability

The complete Si-ZEO22 data set for the 219 zeolite topologies considered in this work and example scripts for accessing the data set are hosted at https://github.com/kul-group/Si-ZEO22.

## Supporting Information Available

DP hyperparameter tuning and learning curves (Tables S1–S5 and Figures S1–S5) and relative training times per 1000 epochs for different hyperparameter combinations (Table S6) (PDF)
Tabulated results for DFT/DP calculations and training set sizes (SiZEO22-results.xlxs), the final DP model (SiZEO22.pb) with the corresponding DeePMD-kit training parameters (SiZEO22-parameters.json), VASP INCAR with the DFT parameters used for generating the Si-ZEO22 data set, and examples of our LAMMPS input (nvt.lammps) and data files (cha.data) (ZIP)
