# Supplementary material for: Predicting Structural Properties of Pure Silica Zeolites Using Deep Neural Network Potentials
Source: J Phys Chem C Nanomater Interfaces. 2023 Jan 13;127(3):1455–63. doi: 10.1021/acs.jpcc.2c08429 (PMC9885523; doi:10.1021/acs.jpcc.2c08429)
Supplement: Supplementary file 1 — jp2c08429_si_001.pdf [file jp2c08429_si_001.pdf]

Supporting Information

Predicting Structural Properties of Pure Silica Zeolites Using Deep Neural Network Potentials

Tyler Sours and Ambarish Kulkarni*

Department of Chemical Engineering, University of California, Davis, Davis 95616, California, United States

Hyperparameter Tuning

The DP training parameters were adjusted to achieve an architecture that balances the accuracy of the energy and force predictions as well as the time to train and evaluate new configurations. The primary parameters we considered for tuning were the size of the embedding neural net, size of the fitting neural net, atomic cutoff radius, initial learning rate, and the number of training steps (Tables and Figures S1-S5, respectively). Other "smaller" parameters (e.g., batch size and decay rate) were found to have a negligible effect on prediction accuracy and thus have been omitted. It was discovered that DeePMD-kit provides a very robust platform that produces reasonable results for most architectures chosen; however, it is still advantageous to tune the parameters to find the least complex architecture necessary to achieve the desired accuracy in the interest of reducing computational expense.

Table S1. Model hyperparameters for tuning the size of the embedding neural net.

Model	**Embedding Net**	Fitting Net	Cutoff	Initial Learning Rate	Training Steps
A	**(8, 16, 32)**	(64, 64, 64)	6.0	0.0005	1×10^6
B	**(16, 32, 64)**	(64, 64, 64)	6.0	0.0005	1×10^6
C	**(32, 64, 128)**	(64, 64, 64)	6.0	0.0005	1×10^6
D	**(32, 64, 128)**	**(128, 128, 128)**	6.0	0.0005	1×10^6

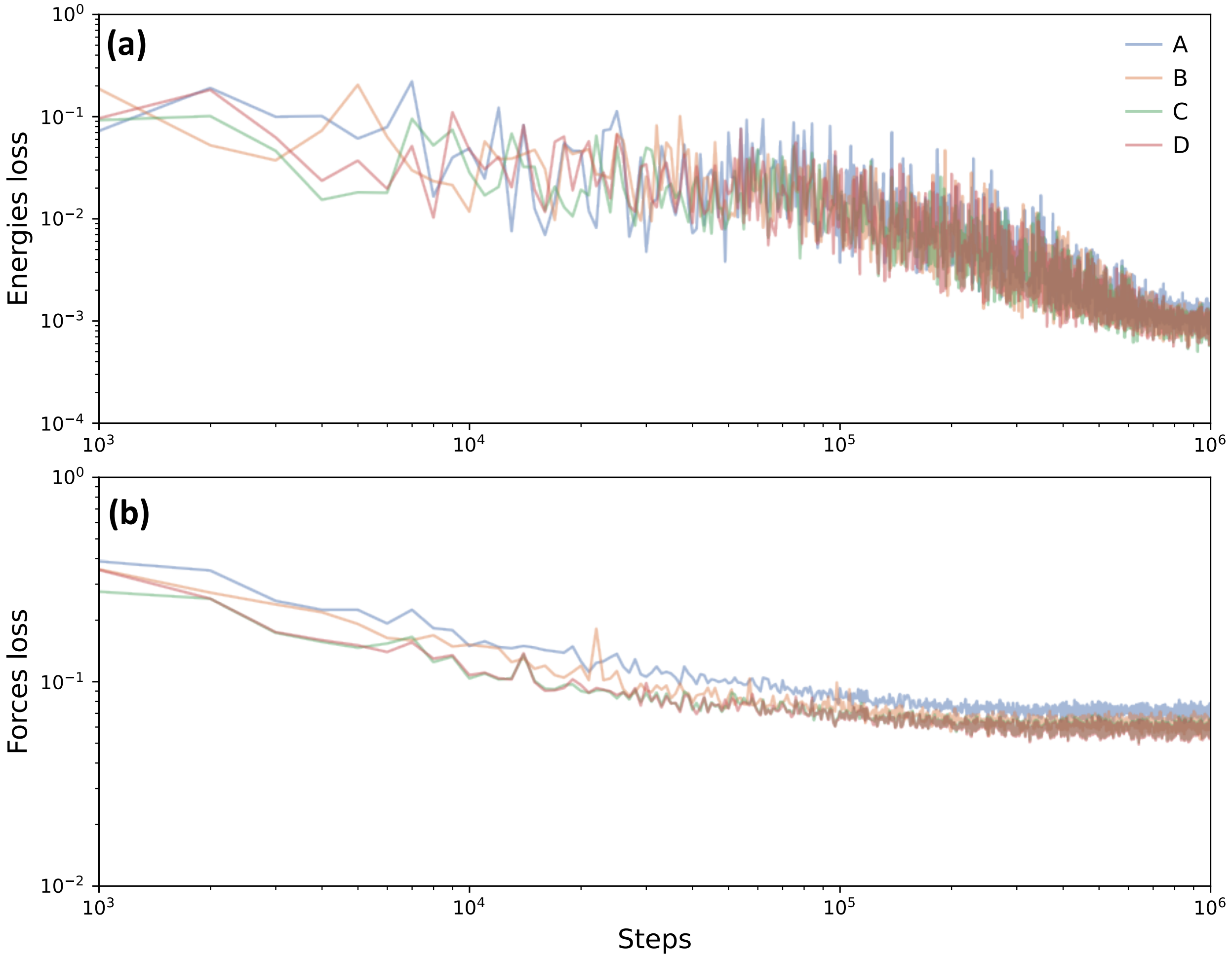

Figure S1. Validation set learning curves for the model hyperparameter combinations shown in Table S1 for the (a) energy and (b) force loss function contributions.

Table S2. Model hyperparameters for tuning the size of the fitting neural net.

Model	Embedding Net	**Fitting Net**	Cutoff	Initial Learning Rate	Training Steps
A	(16, 32, 64)	**(32, 32, 32)**	6.0	0.0005	1×10^6
B	(16, 32, 64)	**(64, 64, 64)**	6.0	0.0005	1×10^6
C	(16, 32, 64)	**(128, 128, 128)**	6.0	0.0005	1×10^6

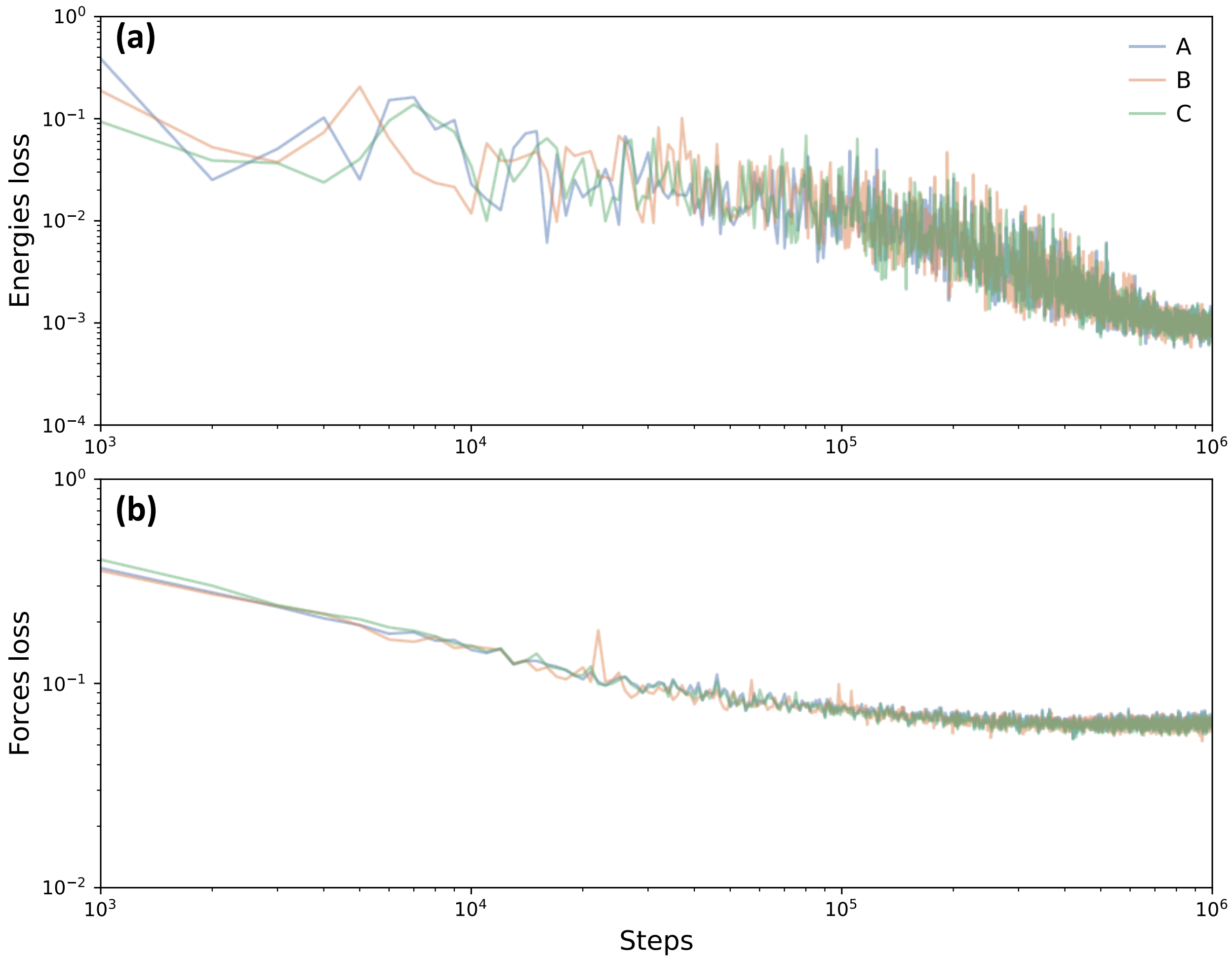

Figure S2. Validation set learning curves for the model hyperparameter combinations shown in Table S2 for the (a) energy and (b) force loss function contributions.

Table S3. Model hyperparameters for tuning the cutoff.

Model	Embedding Net	Fitting Net	**Cutoff**	Initial Learning Rate	Training Steps
A	(16, 32, 64)	(64, 64, 64)	**5.0**	0.0005	1×10^6
B	(16, 32, 64)	(64, 64, 64)	**6.0**	0.0005	1×10^6
C	(16, 32, 64)	(64, 64, 64)	**7.0**	0.0005	1×10^6

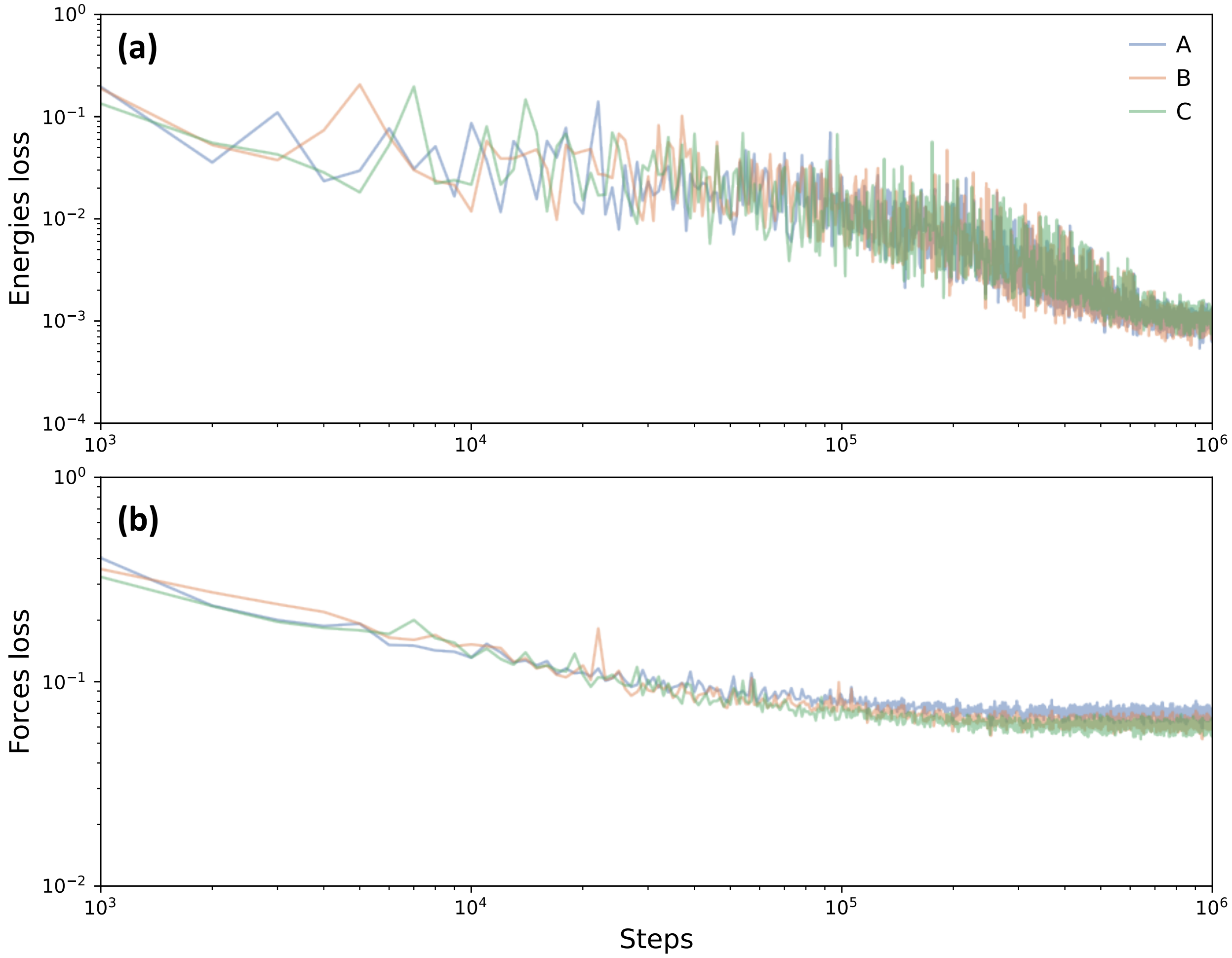

Figure S3. Validation set learning curves for the model hyperparameter combinations shown in Table S3 for the (a) energy and (b) force loss function contributions.

Table S4. Model hyperparameters for tuning the initial learning rate.

Model	Embedding Net	Fitting Net	Cutoff	**Initial Learning Rate**	Training Steps
A	(16, 32, 64)	(64, 64, 64)	6.0	**0.001**	1×10^6
B	(16, 32, 64)	(64, 64, 64)	6.0	**0.0005**	1×10^6
C	(16, 32, 64)	(64, 64, 64)	6.0	**0.0001**	1×10^6

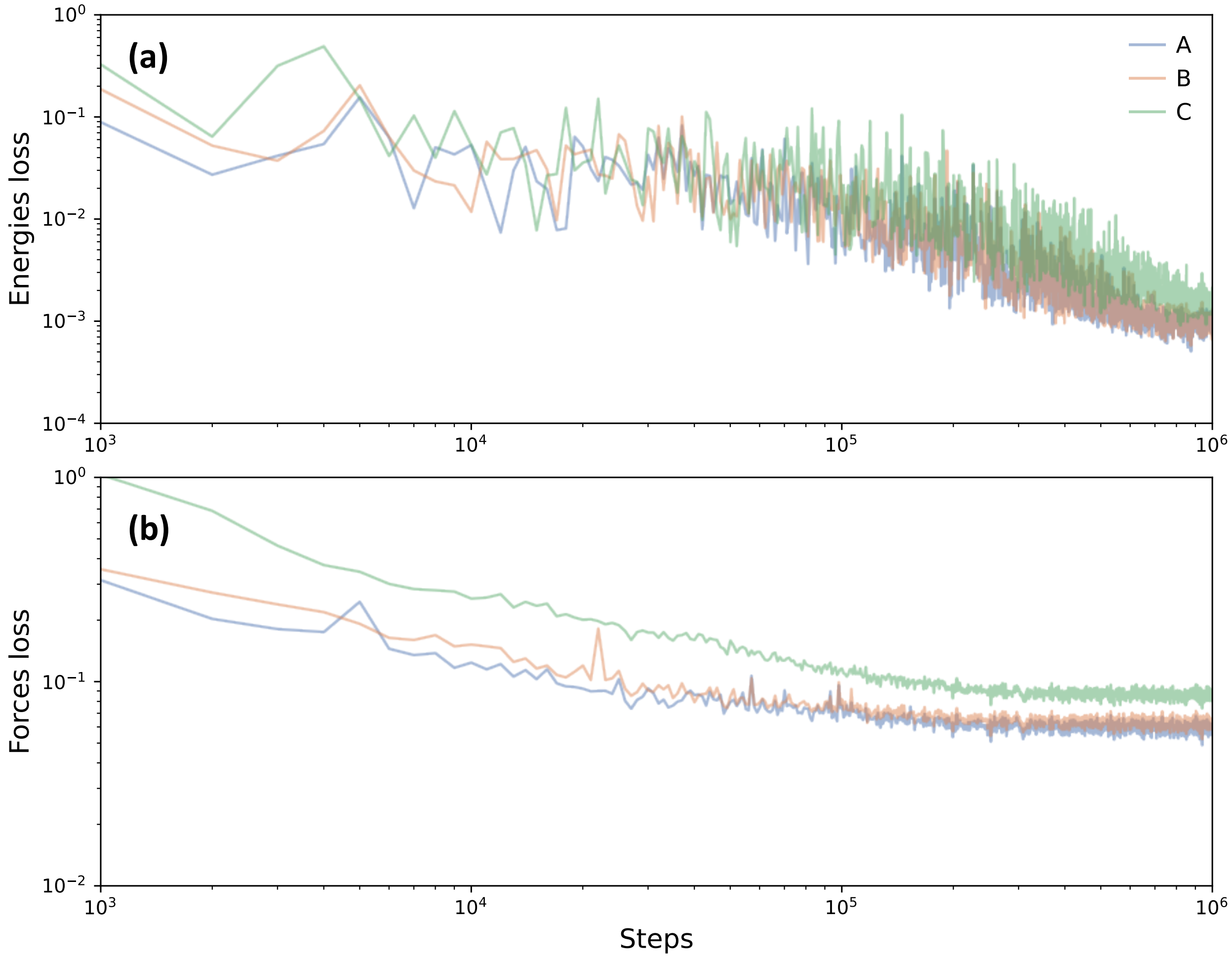

Figure S4. Validation set learning curves for the model hyperparameter combinations shown in Table S4 for the (a) energy and (b) force loss function contributions.

Table S5. Model hyperparameters for tuning the number of training steps.

Model	Embedding Net	Fitting Net	Cutoff	Initial Learning Rate	**Training Steps**
A	(16, 32, 64)	(64, 64, 64)	6.0	0.0005	$\mathbf{5 \times 10^5}$
B	(16, 32, 64)	(64, 64, 64)	6.0	0.0005	$\mathbf{1 \times 10^6}$
C	(16, 32, 64)	(64, 64, 64)	6.0	0.0005	$\mathbf{5 \times 10^6}$
D	(16, 32, 64)	(64, 64, 64)	6.0	0.0005	$\mathbf{2 \times 10^7}$

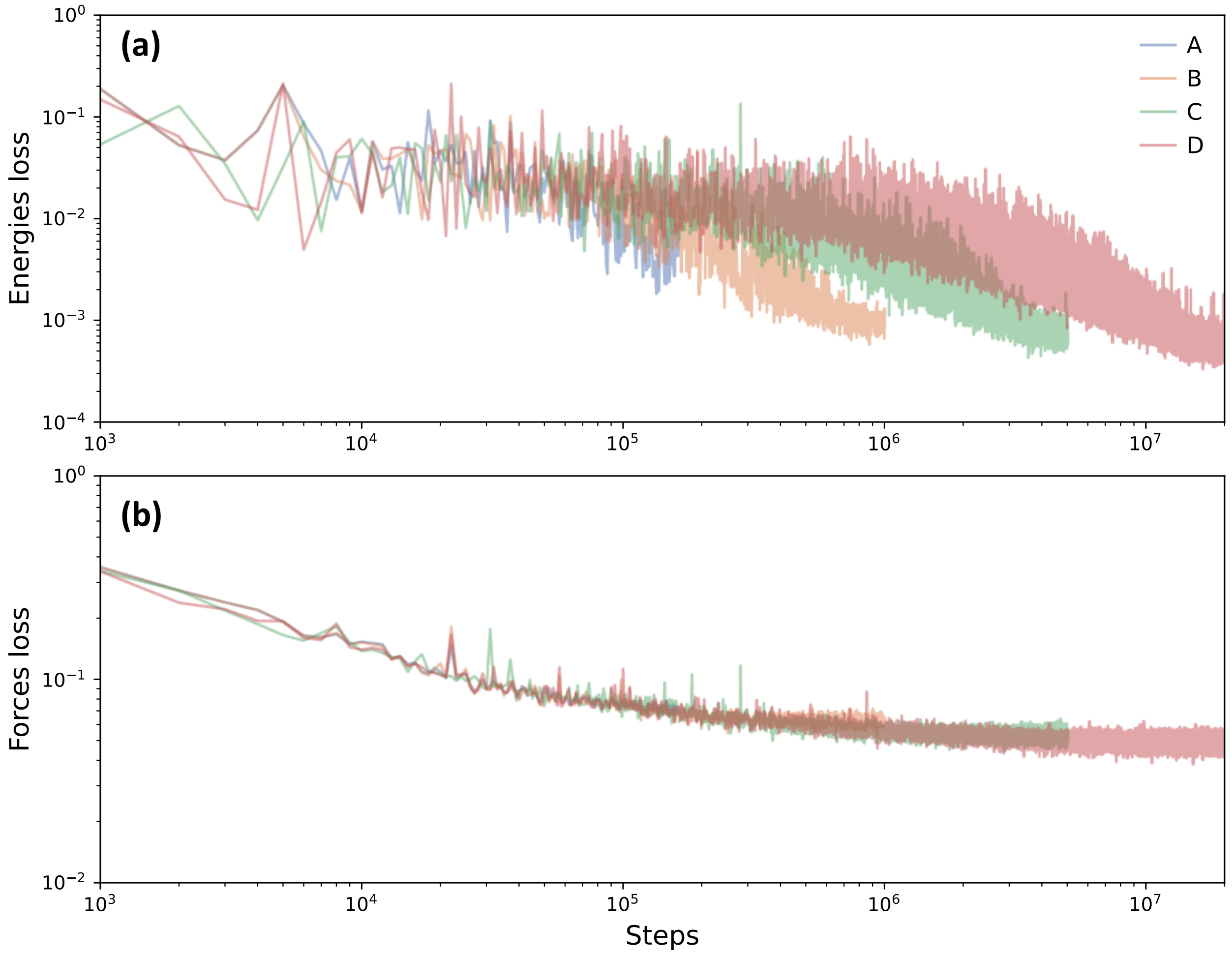

Figure S5. Validation set learning curves for the model hyperparameter combinations shown in Table S5 for the (a) energy and (b) force loss function contributions.

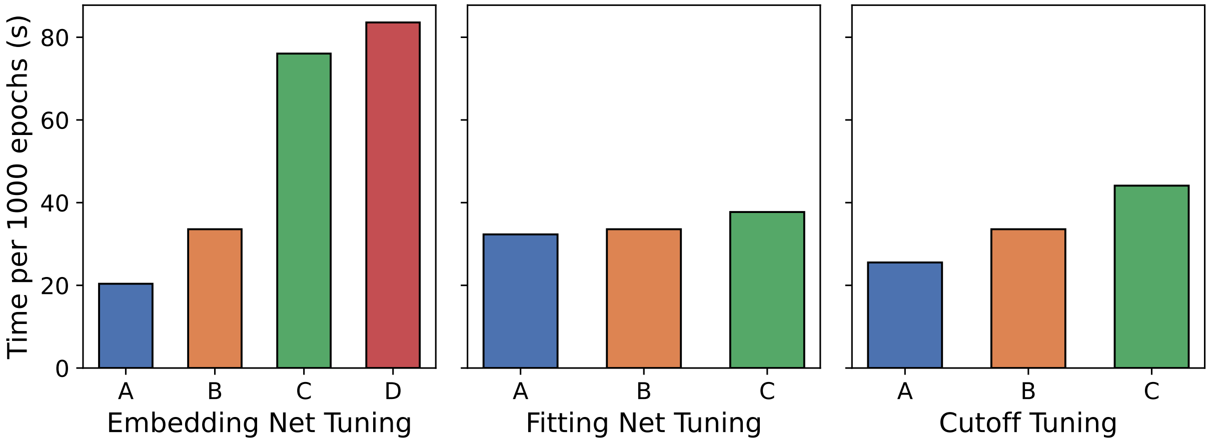

Figure S6. Relative training times per 1,000 epochs for different hyperparameter combinations for tuning the (a) embedding neural net (Table S1), (b) fitting neural net (Table S2), and (c) cutoff (Table S3).
